# Transcriptome Insights into Protective Mechanisms of Ferroptosis Inhibition in Aortic Dissection

**DOI:** 10.3390/ijms26094338

**Published:** 2025-05-02

**Authors:** Chun-Che Shih, Chi-Yu Chen, Chih-Pin Chuu, Chun-Yang Huang, Chia-Jung Lu, Hsin-Ying Lu

**Affiliations:** 1 Department of Surgery, School of Medicine, College of Medicine, Taipei Medical University, 250 Wuxing Street, Taipei 11031, Taiwan; ccshih0603@tmu.edu.tw (C.-C.S.); carollualm@gmail.com (C.-J.L.); 2 Division of Cardiovascular Surgery, Department of Surgery, Wan Fang Hospital, Taipei Medical University, Taipei 11031, Taiwan; 3 Taipei Heart Institute, Taipei Medical University, Taipei 11031, Taiwan; 4 Department of Ophthalmology, Louis J. Fox Center for Vision Restoration, University of Pittsburgh School of Medicine, Pittsburgh, PA 15213, USA; formoxa@gmail.com; 5 Institute of Cellular and System Medicine, National Health Research Institutes, Miaoli 35053, Taiwan; cpchuu@nhri.edu.tw; 6 Department of Medicine, School of Medicine, National Yang-Ming Chiao-Tung University, Taipei 11221, Taiwan; chyhaung@hotmail.com; 7 Division of Cardiovascular Surgery, Department of Surgery, Taipei Veterans General Hospital, Taipei 11217, Taiwan; 8 Division of Cardiovascular Surgery, Department of Surgery, National Yang-Ming Chiao-Tung University Hospital, Yilan 26058, Taiwan; 9 Department of Physical Medicine and Rehabilitation, Wan Fang Hospital, Taipei Medical University, Taipei 11031, Taiwan

**Keywords:** aortic dissection, ferroptosis, inflammation, transcriptomics

## Abstract

Aortic dissection (AD) is a life-threatening vascular condition with limited pharmacological options, and shared risk factors with cardiac disease include hypertension, atherosclerosis, smoking, and dyslipidemia. This study investigated Ferrostatin-1 (Fer-1), a ferroptosis inhibitor, in a BAPN/Ang-II-induced mouse model of AD, revealing significant therapeutic potential. Fer-1 significantly reduced AD incidence and mortality by preserving aortic wall integrity. RNA sequencing identified 922 differentially expressed genes, with 416 upregulated and 506 downregulated. Bioinformatics analysis revealed that Fer-1 modulates key regulators, such as *MEF2C* and *KDM5A*, impacting immune responses, oxidative stress, apoptosis, and lipid metabolism. Additionally, Fer-1 alters miRNA expression, with the upregulation of miR-361-5p and downregulation of miR-3151-5p, targeting pathways involved in inflammation, oxidative stress, and smooth muscle cell (SMC) phenotypic stability. Functional pathway analysis highlighted the inhibition of actin cytoskeleton, *ILK*, and *IL-17* signaling, essential for SMC differentiation and extracellular matrix remodeling. Gene interaction network analysis identified 21 central molecules, including *CXCR3*, *ACACA*, and *BPGM*, associated with lipid metabolism, inflammation, and vascular remodeling. This research elucidates the mechanism of ferroptosis in AD pathogenesis and establishes Fer-1 as a promising therapeutic intervention. AD and cardiac diseases share molecular mechanisms, risk factors, and pathological processes, positioning AD within the broader scope of cardiovascular pathology. By attenuating lipid peroxidation, oxidative stress, and inflammation, Fer-1 may have cardioprotective effects beyond AD, providing a foundation for future translational research in cardiovascular medicine.

## 1. Introduction

Aortic dissection (AD) is a life-threatening vascular condition characterized by the separation of the aortic wall layers, resulting in severe complications, such as rupture and organ ischemia [1]. Despite significant advancements in diagnostic imaging and surgical management, the limited understanding of AD pathogenesis hampers the development of effective early-stage intervention strategies [2,3]. Beyond its vascular consequences, AD profoundly affects cardiac function by altering hemodynamics, imposing increased left ventricular afterload, and compromising coronary perfusion, potentially leading to myocardial ischemia [4]. In Type A dissections, serious complications are initially present or develop subsequently [5]. Additionally, disruption of aortic root integrity can impair coronary artery flow, increasing the risk of acute myocardial infarction. Severe cases may also lead to pericardial tamponade, a fatal complication where blood accumulates in the pericardial sac, restricting cardiac filling and reducing cardiac output, further linking AD to critical heart dysfunction.

Recent studies have identified oxidative stress, inflammation, smooth muscle cell (SMC) phenotypic transition and extracellular matrix (ECM) degradation as critical contributors to AD progression [1,6,7]. These pathological processes frequently culminate in cell death, further compromising the structural integrity of the aortic wall [8]. Although certain agents targeting apoptosis or autophagic cell death have demonstrated protective effects in mouse models of AD, their clinical translation has yet to be achieved. These findings emphasize the pivotal role of regulated cell death in AD pathogenesis and highlight the potential of targeting these pathways as a promising therapeutic strategy [9].

Among the various forms of regulated cell death, ferroptosis—an iron-dependent mechanism characterized by lipid peroxidation—has emerged as a critical factor in vascular injury and inflammation [9,10]. Molecularly, ferroptosis is regulated by key pathways, including the system Xc−/GSH/GPX4 axis, iron metabolism (transferrin receptor, ferritin, heme oxygenase-1), lipid metabolism (ACSL4, LPCAT3), and emerging epigenetic modulators [11]. Ferroptosis has been implicated in the pathogenesis of several cardiovascular diseases, including doxorubicin-induced cardiotoxicity [12], ischemia-reperfusion injury [13], heart failure [14], AD [9,15] and stroke [16]. In AD, elevated oxidative stress creates an environment conducive to ferroptotic cell death, which may exacerbate structural damage to the aortic wall [10]. Recent studies also suggest that ferroptosis may impair SMC viability and contractility, promote endothelial dysfunction, and aggravate ECM breakdown through lipid peroxidation-mediated inflammation and metalloproteinase activation [17,18]. However, the interplay between ferroptosis and other pathological processes in AD, such as smooth muscle cell dysfunction and ECM degradation, remains inadequately explored [9,15,19]. This knowledge gap underscores the critical need for focused investigations into ferroptosis-mediated mechanisms and their potential therapeutic modulation in AD.

To better understand the therapeutic potentials of targeting ferroptosis, we have examined the effects of Fer-1, a potent ferroptosis inhibitor, in β-aminopropionitrile (BAPN) and angiotensin II (Ang-II)-induced AD mouse models. Fer-1 has demonstrated efficacy in preclinical models of oxidative stress-related diseases by scavenging lipid peroxides and preserving cellular antioxidant defenses. These mechanisms effectively mitigate oxidative damage and inflammation [9,20,21]. This study aims to elucidate the molecular mechanisms underlying the therapeutic effects of Fer-1 and its role in inhibiting ferroptosis through histological, transcriptomic, and pathway analyses.

## 2. Results

### 2.1. Treatment with Fer-1 Attenuated AD Development in Mice

**Figure 1:**
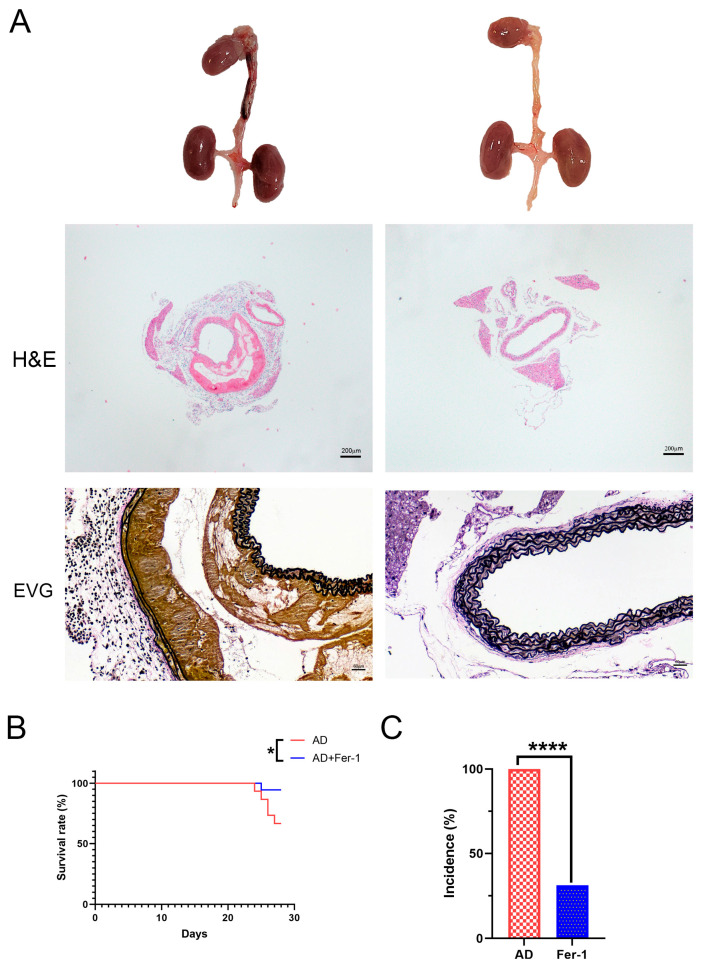
Fer-1 ameliorates the etiopathology of BAPN/Ang-II-induced AD in mice. (**A**) Representative morphologies of aortas, H and E, and VVG staining in aorta. (**B**) Incidence. (**C**) Mortality. The data are expressed as mean ± SD. Statistical significance: * *p* < 0.05, **** *p* < 0.0001.

Mice treated with Fer-1 showed a marked reduction in the development and progression of AD. Histological analysis further supported these findings, showing preserved aortic wall integrity and reduced structural damage in Fer-1-treated mice (Figure 1A). This therapeutic effect was evident in both the decreased incidence of AD (Figure 1B) and improved survival rates (Figure 1C) compared to the untreated AD group. These results suggest that Fer-1 plays a protective role in AD development.

### 2.2. Identification of Differentially Expressed Genes (DEGs) After Fer-1 Treatment in AD Mice

**Figure 2:**
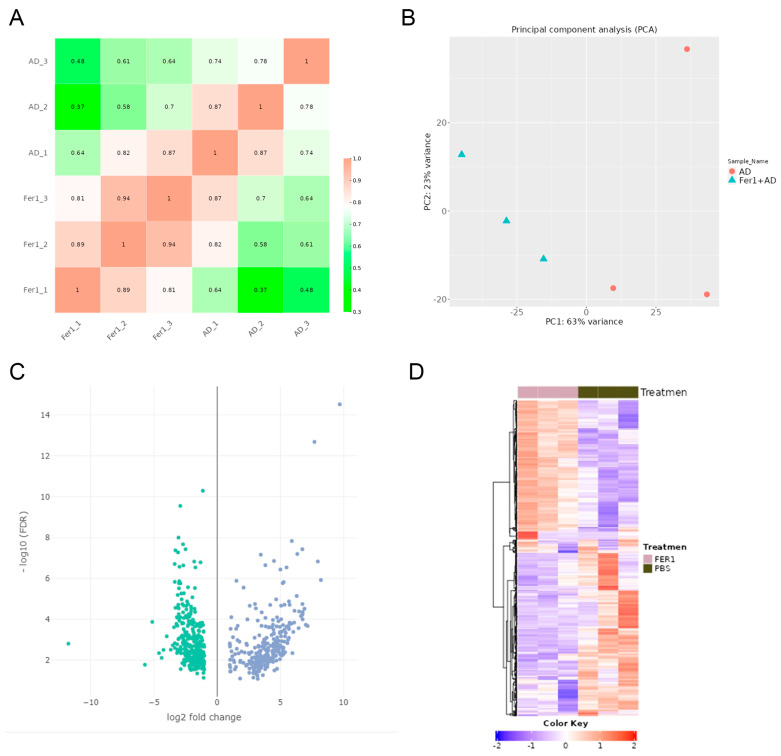
Differentially expressed genes (DEGs) in Fer-1-treated aorta compared with AD controls. (**A**) Heatmap correlation among the samples. The color scale indicates the values of the correlations. (**B**) Principal component analysis (PCA) of RNA-seq dataset. (**C**) Volcano plot displaying DEGs under the cut-off: *p* value < 0.05 and absolute log2FoldChange > 0.58. Green dots: significantly downregulated genes; blue dots: significantly upregulated genes. (**D**) Heatmap of DEGs.

To evaluate the consistency of gene expression among the samples, a correlation heatmap was generated, revealing strong positive correlations with no outlier samples (Figure 2A). Principal component analysis (PCA) further supported these findings, showing a clear separation between Fer-1-treated and AD control groups. Samples within each group formed distinct clusters, indicating high reproducibility and consistency in gene expression patterns (Figure 2B).

922 DEGs were identified when comparing Fer-1-treated aortic samples to AD control samples. Of these, 416 genes were upregulated, while 506 were downregulated (Figure 2C,D). The top 10 upregulated and downregulated genes are summarized in Table 1, highlighting key transcriptional changes induced by Fer-1 treatment.

These findings will provide a comprehensive overview of the transcriptional alterations associated with Fer-1 treatment and offer a foundation for further investigation into the molecular mechanisms underlying its protective effects in AD.

### 2.3. Upstream Regulators of Differential Gene Expression

Upstream regulators, including transcription factors, cytokines, small RNAs, receptors, kinases, chemical molecules, and pharmacological agents, influence gene expression patterns. Using a threshold of overlap *p*-value < 0.05, 1728 upstream regulators were identified. Among these, 93 were significantly activated (activation Z-score > 2), and 270 were significantly inhibited (activation Z-score < −2). The top 15 activated and inhibited upstream regulators are listed in Table 2.

**Figure 3:**
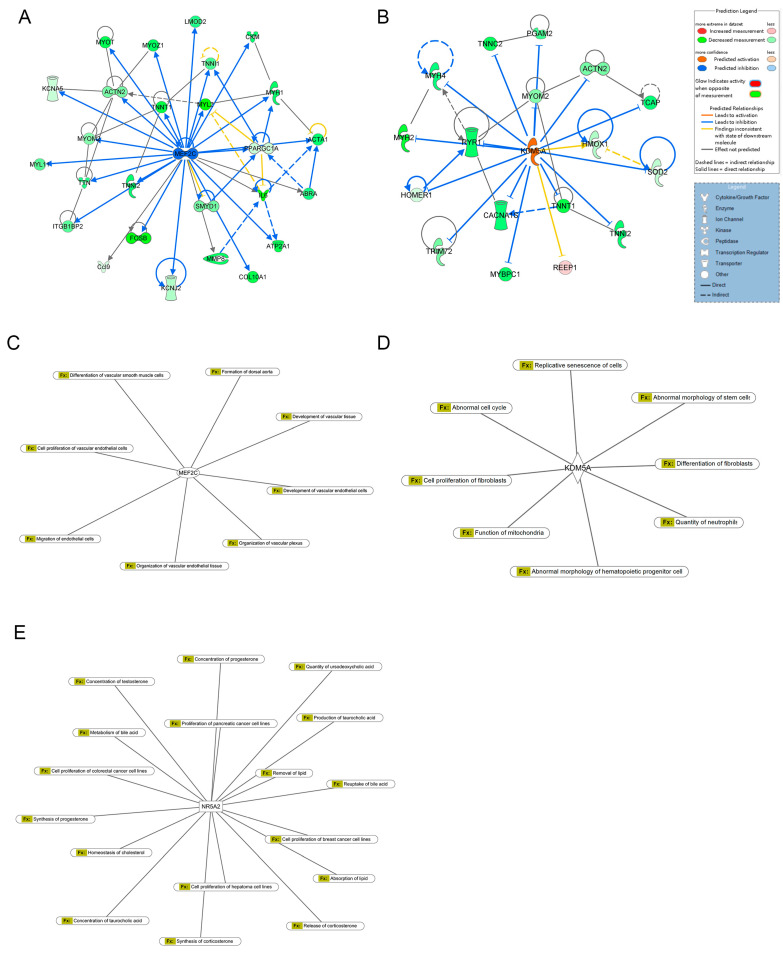
Upstream regulator analysis of DEGs. (**A**) Downstream targets of MEF2C among DEGs. (**B**) Downstream targets of KDM5A among DEGs. (**C**) The diseases and biofunctions were regulated by MEF2C. (**D**) The diseases and biofunctions were regulated by KDM5A. (**E**) The diseases and biofunctions were regulated by NR5A2.

MEF2C was identified as the most potent inhibitor (Z-score = −4.358; overlap *p*-value = 3.06 × 10^−18^), regulating 29 enriched target genes, including *ABRA*, *ACTA1*, *ACTN2*, *ATP2A1*, *Ccl9*, *CKM*, *COL10A1*, *CXCL2*, *CXCL6*, *FOSB*, *IL6*, *ITGB1BP2*, *KCNA5*, *KCNJ2*, *LMOD2*, *MMP8*, *MYH1*, *MYH7*, *MYL11*, *MYL2*, *MYOM2*, *MYOT*, *MYOZ1*, *PPARGC1A*, *SMYD1*, *TNNI1*, *TNNI2*, *TNNT1*, *TTN* (Figure 3A). In contrast, *KDM5A* emerged as the most robust activator (Z-score = 3.441; overlap *p*-value = 3.71 × 10^−9^), targeting 18 genes, including *ACTN2*, *Actn3*, *CACNA1S*, *HMOX1*, *HOMER1*, *MYBPC1*, *MYH2*, *MYH4*, *MYH7*, *MYOM2*, *PGAM2*, *REEP1*, *RYR1*, *SOD2*, *TCAP*, *TNNC2*, *TNNI2*, *TNNT1*, *TRIM72* (Figure 3B). In-depth analysis of DEGs revealed that MEF2C primarily regulates genes associated with vascular cell functions and vascular development (Figure 3C). *KDM5A* modulated genes involved in cellular homeostasis (Figure 3D). Additionally, *NR5A2* was linked to the regulation of lipid metabolism (Figure 3E).

**Figure 4:**
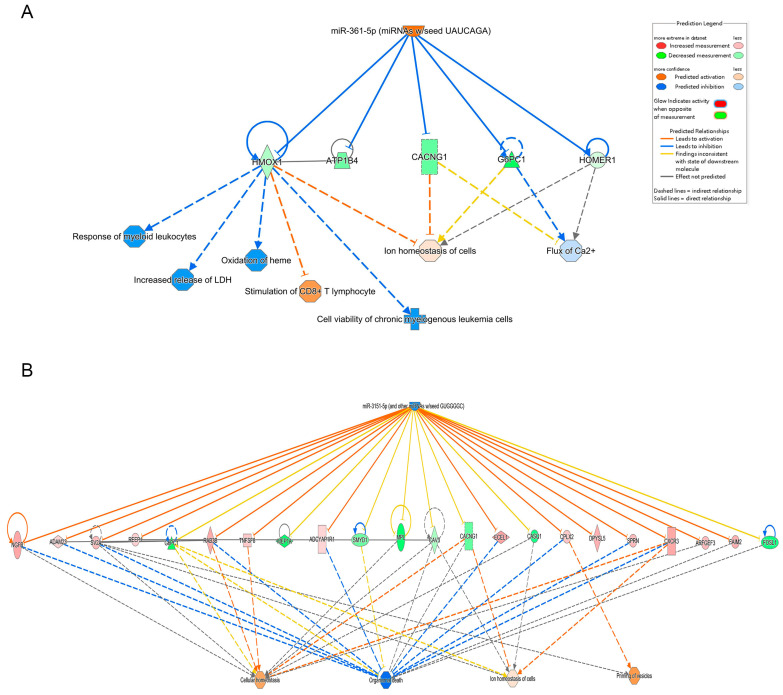
Integrated pathway analysis of upstream molecules, gene expression and function predictions. (**A**) miR-361, (**B**) miR-3151-5p. The top row shows each miRNA, the middle row shows the gene predicted to be regulated by each miRNA and the bottom row shows the function predicted to be involved. Orange miRNAs are upregulated, blue miRNAs are downregulated, peach genes are upregulated, green genes are downregulated and orange predicted functions are upregulated. Arrows indicate predicted relationships: orange arrows represent activation, blue arrows represent inhibition, yellow arrows indicate inconsistency with the downstream molecule’s state, and gray arrows represent no predicted effect. Solid lines denote direct relationships, while dashed lines represent indirect relationships.

Differential miRNA expression analysis identified 122 miRNAs with altered expression in the Fer-1-treated group compared to the AD group (Table S1). Using an absolute Z-score > 2 as the threshold, the most significantly upregulated and downregulated miRNAs were highlighted. Their regulatory networks, including target genes, are illustrated in Figure 4, providing insights into their roles in modulating the observed transcriptional changes.

These findings underscore the complex regulatory mechanisms driving the effects of Fer-1 treatment, particularly in vascular development, homeostasis, and lipid metabolism.

### 2.4. Functional Prediction and Enriched Canonical Pathways

To understand the biological implications of DEGs, we employed multiple analytical approaches via IPA software for comprehensive canonical pathway investigation.

**Figure 5:**
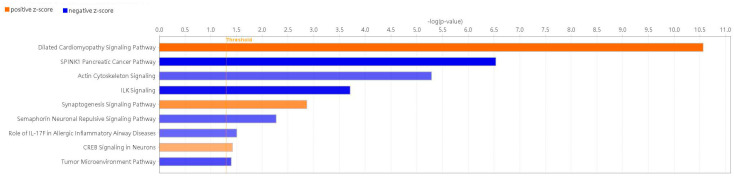
Enriched canonical pathways of DEGs. Blue band: the negative prediction of the pathway; orange band: the active prediction of the pathway. The filter was absolute z-score > 2.0. The threshold line was drawn at −log(*p*-value) = 1.3.

IPA revealed 145 enriched canonical pathways under the significance threshold of −log(*p*-value) > 1.3, with 72 pathways meeting the additional criteria of −log(*p*-value) > 1.3 and |z-score| ≥ 0.0 as shown in Table S2. By applying a significance threshold of an absolute z-score greater than 2.0, we identified several key findings s sown in Figure 5. Pathways such as ‘Dilated Cardiomyopathy Signaling Pathway’ (z-score = 3.873), the ‘Synaptogenesis Signaling Pathway’ (z-score = 2.840), and the ‘CREB Signaling in Neurons’ (z-score = 2.132) showed significant activation. Pathways like ‘SPINK1 Pancreatic Cancer Pathway’ (z-score = −3.317), the ‘ILK Signaling’ (z-score =−3.051), the ‘Tumor Microenvironment Pathway’ (z-score = −2.333), the ‘Actin Cytoskeleton Signaling’ (z-score = −2.138), the ‘Semaphorin Neuronal Repulsive Signaling Pathway’ (z-score = −2.121) and ‘Role of IL-17F in Allergic Inflammatory Airway Diseases’ (z-score = −2.000) demonstrated significant inhibition. Additional specific signaling pathways associated with AD, including ‘IL-17’, ‘ferroptosis’, ‘cytoskeleton signaling’ and ‘macrophage classical activation signaling pathways’, are shown in Figures S1–S4.

These analyses provide valuable insights into the complex biological functions and pathways modulated by Fer-1, shedding light on its potential therapeutic mechanisms.

### 2.5. Disease and Biofunction Analysis

**Figure 6:**
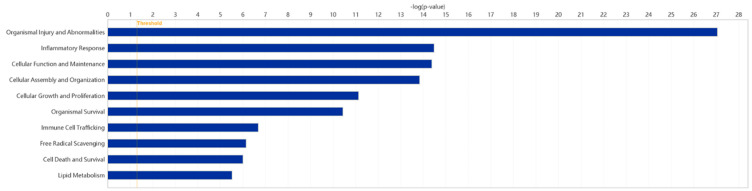
Enriched diseases and bio functions of DEGs. A total of 10 representative classification of diseases and functions possibly mediated by Fer-1 are plotted. The threshold line was drawn at −log(*p*-value) = 1.3.

Using the IPA system with a significance threshold of −log(*p*-value) > 4, the role of Fer-1 in disease and cellular functions was analyzed. The analysis revealed key classifications of disease and disorders, molecular and cellular functions, and physiological system development and functions, summarized in a histogram (Figure 6). Fer-1 was found to play significant roles in cellular functions relevant to AD, particularly in processes associated with inflammation and vascular dynamics. Notable functions modulated by Fer-1 included the “Response of phagocytes” (−log(*p*-value) = 5.769, z-score = −1.836), indicating an inhibitory effect on phagocyte-related responses. Similarly, Fer-1 was linked to the “Occlusion of blood vessels” (−log(*p*-value) = 5.635, z-score = −1.93), further suggesting its regulatory impact on vascular integrity. Additional associations were identified with “Inflammation of organs” (−log(*p*-value) = 10.578, z-score = −0.316) and the “Synthesis of reactive oxygen species” (−log(*p*-value) = 6.142, z-score = −0.087), processes integral to oxidative stress and inflammatory responses (Figures S5–S8).

These findings highlight Fer-1’s multifaceted role in modulating cellular responses related to AD, emphasizing its potential as a therapeutic agent targeting inflammatory and vascular dysfunctions.

### 2.6. Interaction Network Analysis

The interaction network analysis identified key molecular interactions within the dataset, providing insights into functional relationships. Networks were ranked based on score values, with detailed results provided in Table S3. The highest-ranked network, with a score of 33, was primarily associated with “Cardiovascular Disease, Cell Death and Survival, and Cellular Assembly and Organization”. This network included 21 molecules from the DEGs dataset: *ACACA*, *ASB5*, *BPGM*, *C11orf54*, *Ces2c*, *CLVS2*, *CLYBL*, *CXCR3*, *DISP2*, *DZANK1*, *ENO1*, *ENO2*, *EYA1*, *GIPC2*, *Gypa*, *GZMA*, *HEMGN*, *HNF4A*, *IFI16*, *IGF2BP2*, and *Ldh*.

**Figure 7:**
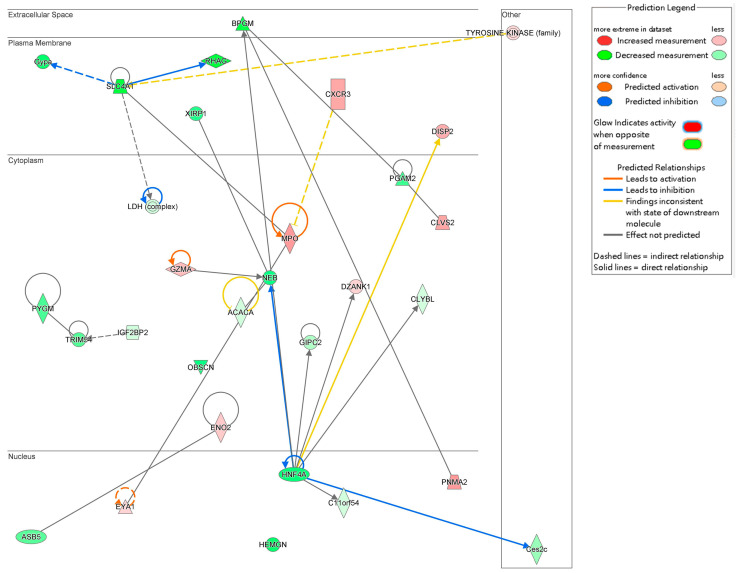
Gene interaction network map. This network consists of the top ranked network found associated with the role of Fer-1 treatment in cardiovascular disease, cell death and survival, cellular assembly and organization. Arrows indicate predicted relationships: orange arrows represent activation, blue arrows represent inhibition, yellow arrows indicate inconsistency with the downstream molecule’s state, and gray arrows represent no predicted effect. Solid lines denote direct relationships, while dashed lines represent indirect relationships.

The interactions among these 21 DEGs are visualized in Figure 7, illustrating their complex relationships and potential cooperative roles in cardiovascular disease mechanisms, cell survival processes, and cellular organization. These findings underscore the importance of these molecular networks in the context of Fer-1 treatment and its therapeutic implications.

## 3. Discussion

Ferroptosis, characterized by iron-dependent lipid peroxidation, has emerged as a central mechanism in vascular injury. The histological analyses in this study demonstrated that Fer-1 preserved aortic wall integrity in a BAPN/Ang-II-induced AD mouse model. The observed reduction in AD incidence and mortality highlights the critical role of ferroptosis in early structural damage and disease progression. These findings align with previous reports indicating that increased oxidative stress and inflammation create a favorable environment for ferroptosis in AD [15,19,22].

Gene expression analysis identified key upstream regulatory molecules influenced by Fer-1 treatment. *MEF2C* and *KDM5A* were identified as potential regulators of AD pathogenesis. MEF2C is known to protect against atherosclerosis by inhibiting TLR/NF-κB activation, SMC migration [23], and proliferation [24,25]. It regulates KLF2, which enhances endothelial barrier function and inhibits atherosclerosis, thrombosis, and coronary artery lesions [23,26,27]. Hyper-uric acid exerts thrombogenic effects in mice by upregulating let-7c and activating the NF-κB pathway in a MEF2C-dependent manner [28]. MEF2C overexpression mitigates apoptosis in cerebral ischemia preconditioning and suppresses inflammation and oxidative stress by inhibiting NF-κB phosphorylation [29,30]. Additionally, MEF2C alleviates postoperative cognitive dysfunction by repressing ferroptosis [31]. KDM5A, another identified regulator, maintains genomic integrity and modulates key processes, such as cell cycle, apoptosis, and metabolism [32,33]. KDM5A plays a pivotal role in vascular VSMC homeostasis, regulating proliferation, differentiation, and vascular remodeling. Loss of KDM5A function disrupts VSMC homeostasis, increasing susceptibility to AD [34]. Additionally, another upstream regulator, NR5A2, identified in our datasets, is involved in lipid metabolism. NR5A2 plays critical roles in embryonic development, cholesterol and bile acid homeostasis, and cell proliferation [35]. NR5A2 has also been shown to indirectly regulate the immune system and associated inflammatory processes via the synthesis of immunoregulatory glucocorticoids in the intestinal crypts [36]. Tissue-specific deletion or inhibition of NR5A2 and associated intestinal glucocorticoid synthesis consequently results in increased susceptibility to the development of intestinal inflammatory disorders [37]. Based on our analysis, the upstream regulators *MEF2C*, *KDM5A*, and *NR5A2*, along with the downregulation of their target genes (*Ccl9*, *COL10A1*, *CXCL2*, *MMP8*, *Ccl7*, *IL1B*, *IL6*, *SOD2*, and others), may represent an additional mechanism by which Fer-1 exerts its therapeutic effects in AD. This mechanism likely involves the modulation of immune responses, oxidative stress, apoptosis, and lipid metabolism, as well as the prevention of inappropriate phenotypic alterations in SMCs and the enhancement of endothelial barrier function.

In addition to epigenetic regulation, miRNAs were also implicated in the protective effects of Fer-1. MiR-361-5p was previously associated with acute coronary syndrome and endothelial cell function [38,39]. The expression of miR-361-5p was significantly decreased in ox-LDL injured vascular SMCs, while lncRNA MEG3-derived miR-361-5p regulate vascular SMC proliferation and apoptosis by targeting ABCA1 [40]. In this study, miR-361-5p was predicted to be upregulated following Fer-1 treatment. It was identified as an upstream regulator of genes such as *HMOX1*, *ATP1B4*, *CACNG1*, *G6PC1*, and *HOMER1*, which are involved in modulating oxidative stress, inflammation, and ion homeostasis. Previous research has established a strong association between HMOX1 and ferroptosis [38,41,42], with excessive HMOX1 expression potentially triggering ferroptosis. Conversely, miR-3151-5p expression was found to be downregulated in Fer-1-treated mice. This miRNA is predicted to target genes related to cell death (e.g., *CASP3*, *CASP9*, *FAS*), ion homeostasis (e.g., *CALB1*), and inflammation (e.g., *IL6*, *IL1B*, *NLRP3*). Notably, the downregulation of *IL6* and *IL1B*, key inflammatory mediators, observed in Fer-1-treated mice corresponds with the reduction in inflammation, further supporting the therapeutic effects of Fer-1.

The plasticity of SMCs is crucial for vascular compliance and ECM regulation, particularly in aortic disease pathogenesis. PAI-1 inhibits cofilin, a key cytoskeletal regulator, thereby influencing SMC stiffness and F-actin content [43]. Consistent with our findings, Fer-1 treatment regulated cytoskeleton polymerization by reducing F-actin level. ILK an intracellular serine/threonine kinase, plays a critical role in cell–matrix interactions and signal transduction [44]. It induces AKT phosphorylation and p21 degradation, which are associated with SMC migration and proliferation [45]. Furthermore, ILK activates MMP-9 promoter, and consequently MMP-9 expression through the GSK-3β/AP-1 pathway [46] and contributes to Ang-II-induced renal inflammation [47]. SMOC2 silencing alleviated cardiac fibrosis through inhibition of the ILK/p38 signaling [48]. IL-17 plays a critical role in acute inflammation and is reported to participate in AD pathogenesis [49] by interfering with TGF-β signal and altering ECM metabolism [50]. A critical role for IL-17 in post-myocarditis cardiac remodeling and the progression to dilated cardiomyopathy was also reported [51,52]. Additionally, Fer-1 treatment downregulated pro-inflammatory M1 macrophage pathways while upregulating anti-inflammatory M2 macrophage pathways. Findings from our datasets according to enrichment analysis on canonical pathways of IPA suggest that Fer-1 preserves SMC differentiation and attenuates ECM remodeling through its anti-inflammatory and cytoskeletal regulatory effects.

Gene interaction network analysis further revealed key molecules, such as *CXCR3*, *ACACA*, and *BPGM*, which play roles in inflammation, lipid metabolism, and vascular wall integrity. CXCR3 regulates immune cell recruitment and inflammatory signaling, processes exacerbating aortic wall damage. ACACA, a key enzyme in fatty acid biosynthesis, influences lipid homeostasis, while BPGM reflects metabolic adaptation under oxidative stress. These interactions indicate that Fer-1 not only inhibits ferroptosis but also modulates broader metabolic and inflammatory pathways to support vascular health.

Another insightful pathway in this study identified 922 DEGs associated with ferroptosis inhibition, including pathways related to phagocyte response, occlusion of blood vessel, and ROS synthesis against AD. While this research provides compelling evidence for the role of ferroptosis in AD and the protective effects of Fer-1, certain limitations remain. While our findings offer important insights into the molecular responses to Fer-1 and suggest possible regulatory mechanisms, we acknowledge that these conclusions are based on mRNA-level data and in silico predictions. It is important to emphasize that transcriptomic data alone cannot confirm changes at the protein level, nor can they establish causality in the observed interactions. Functional validation of identified DEGs and their roles in ferroptosis and AD pathogenesis is necessary. Additionally, transgenic animal studies could clarify the contributions of targets, such as *MEF2C* and *KDM5A*. The acute effects of Fer-1 were the primary focus; future studies should investigate its long-term efficacy and potential side effects. Dose-response studies are also needed to optimize therapeutic outcomes. Despite this limitation, we believe that our data provide a valuable resource for guiding future research on the molecular mechanisms of Fer-1 and ferroptosis regulation.

## 4. Materials and Methods

### 4.1. Model Establishment and Interventions

All animal experiments were approved by the Institutional Animal Care and Use Committee of Taipei Medical University. Male C57BL/6J mice were housed in a specific pathogen-free (SPF) facility under a 12-h light–dark cycle and fed a standard diet. An aortic dissection (AD) model was induced in three-week-old mice by oral administration of 0.5% β-aminopropionitrile (BAPN; Sigma-Aldrich, St. Louis, MO, USA) for four consecutive weeks. A subset of mice from each group was implanted subcutaneously with osmotic minipumps (Model 1003D Micro-osmotic Pump; Alzet, Cupertino, CA, USA) delivering angiotensin II (Ang II; 1 mg/kg/min) for the final 48 h of the experiment. The endpoint of the experiment was set at day 28 following BAPN initiation. Mice were randomly assigned into two groups: the AD group (BAPN alone, *n* = 15) and the Fer-1 treatment group (BAPN + Fer-1, *n* = 15). Mice in the Fer-1 group received daily intraperitoneal injections of ferrostatin-1 (Fer-1; 1 mg/kg/day; Sigma-Aldrich) starting from day 7 and continuing through day 28. Control mice received equivalent volumes of vehicle (PBS). Throughout the experiment, mice were monitored daily for survival, and all deaths were recorded. On day 28, all surviving mice were euthanized, and the aorta was harvested for further study.

### 4.2. Histological Analysis

Excised aortic tissues were fixed in 4% formaldehyde overnight, dehydrated, embedded in paraffin, and sectioned into 4 μm slices. These sections were stained using hematoxylin and eosin (H and E), Masson’s trichrome, or elastin Verhoeff-van Gieson (EVG) staining techniques and examined using EasyScan device (Motic China Group, Ltd., Xiamen, China).

### 4.3. RNA Sequencing

Total RNA was extracted from the ascending aorta and the descending thoracic aorta and assessed for quality using the Agilent 2100 Bioanalyzer (Agilent Technologies, CA, USA). Only samples with an RNA integrity number (RIN) greater than 5.5 were used for library preparation. Sequencing library were constructed using TruSeq Stranded mRNA Library Prep Kit (Illumina, San Diego, CA, USA) following the manufacturer’s recommendations. Total RNA (1 μg) was purified to isolate mRNA using oligo (dT)-coupled magnetic beads. Fragmented mRNA was used to synthesize first-strand cDNA via reverse transcription with random primers. Double-stranded cDNA was generated, and 3′ ends were adenylated before adaptor ligation and purification using the AMPure XP system (Beckman Coulter, Beverly, CA, USA). Sequencing was conducted on an Illumina NovaSeq 6000 platform with 150-bp paired-end reads by a commercial sequencing service (Genomics, BioSci and Tech Co., New Taipei City, Taiwan). Raw sequencing reads were subjected to quality control and adapter trimming. Clean reads were aligned to the mouse reference genome (GRCm38/mm10) using HISAT2 (version 2.2.1). Gene expression was quantified and normalized using Fragments Per Kilobase of transcript per Million mapped reads (FPKM) to account for transcript length and sequencing depth. Differential gene expression analysis was performed using the DESeq2 package (version 1.48) in R (version 4.5).

### 4.4. Bioinformatics and Network Analysis

Bioinformatics analyses were conducted using the Ingenuity Pathway Analysis (IPA) software (version 2024, QIAGEN, Redwood City, CA, USA), focusing on canonical pathways, diseases and functions, regulatory effects, upstream regulators, and molecular networks. Specifically, IPA was employed to analyze our differentially expressed genes (DEGs) and predict potential upstream regulators, such as transcription factors, cytokines, and chemical compounds, whose activation or inhibition could explain the observed gene expression patterns. The chemical compound predictions generated by IPA are hypothesis-generating, reflecting known or predicted associations between those compounds and similar gene expression profiles reported in the literature, rather than representing direct measurements from the RNA. Our study indirectly identified epigenetic regulatory signals, including predicted microRNA regulation and upstream modifiers, such as *KDM5 B*, through IPA-based analyses. Additional analyses, including hierarchical clustering (heatmap), principal component analysis (PCA), and volcano plot, were performed using Integrated Differential Expression and Pathway Analysis (iDEP) version 2.01 (http://bioinformatics.sdstate.edu/idep/, accessed on 4 December 2024).

### 4.5. Statistical Analysis

For incidence comparison, Fisher’s exact test was used between groups. For survival analysis, we have replaced the original bar graph with a Kaplan–Meier survival curve, and the comparison between groups was conducted using the log-rank (Mantel–Cox) test. A *p*-value < 0.05 was considered statistically significant. Statistical analyses were performed using the IPA platform and other methods. Pearson’s correlation was employed to assess group correlations. Differentially expressed genes (DEGs) were analyzed using a two-tailed unpaired *t*-test or Fisher’s exact test, with significance thresholds set at fold change (FC) ≥ 1.2 and *p*-value < 0.05. For IPA, results with |z-score| ≥ 2 and/or overlap *p*-value < 0.05 were considered statistically significant.

## 5. Conclusions

This research elucidates the mechanism of ferroptosis in AD pathogenesis and establishes Fer-1 as a promising therapeutic intervention. Given the close relationship between AD and cardiac disease, improving AD outcomes may significantly reduce the incidence of cardiac complications. Preventing or stabilizing AD could help maintain aortic valve function, prevent myocardial ischemia, and reduce the burden of heart failure. By minimizing hemodynamic stress and inflammation, targeted therapies for AD may also contribute to broader cardiovascular protection.

## Figures and Tables

**Table 1:** The top 10 upregulated or downregulated genes by Fer-1 in AD aorta.

Fer-1/PBS							
Symbol	Entrez Gene Name	Function	Fold Change	Symbol	Entrez Gene Name	Function	Fold Change
*Ctrl*	Chymotrypsin-like	Hydrolase, Protease, Serine protease	6.160	*Bpifa1*	BPI fold containing family A member 1	Antibiotic, Antimicrobial	−9.438
*Pnlip*	Pancreatic lipase	Hydrolase	5.944	*Myl2*	Myosin light chain 2	Motor protein, Muscle protein, Myosin	−8.398
*Rnase1*	Ribonuclease, RNase A family, 1	Endonuclease, Hydrolase, Lyase, Nuclease	5.754	*Myh7*	Myosin heavy chain 7	Actin-binding, Calmodulin-binding, Motor protein, Muscle protein, Myosin	−8.184
*Ctrb1*	Chymo-trypsinogen B1	Hydrolase, Protease, Serine protease	5.483	*Scgb1a1*	Secretoglobin family 1A member 1	Phospholipase A2 inhibitor	−7.8139
*Try4*	Serine protease 3	Hydrolase, Protease, Serine protease	5.395	*Cxcl2*	C-X-C motif chemokine ligand 2	Cytokine	−7.309
*Cela2a*	Chymotrypsin like elastase 2A	Hydrolase, Protease, Serine protease	5.379	*Csf3*	Colony stimulating factor 3	Cytokine, Growth factor	−7.091
*Cel*	Carboxyl ester lipase	Hydrolase, Serine esterase	4.822	*Mstn*	Myostatin	Cytokine, Growth factor, Heparin-binding	−6.867
*Cpa2*	Carboxypeptidase A2	Carboxypeptidase, Hydrolase, Metalloprotease, Protease	4.789	*Alas2*	5′-aminolevulinate synthase 2	Acyltransferase, Transferase	−6.860
*Cd40lg*	CD40 ligand	Cytokine	4.0546	*Il6*	Interleukin 6	Cytokine, Growth factor	−6.843
*Klhl14*	Deleted in malignant brain tumors 1	Developmental protein	3.869	*Fosb*	FosB proto-oncogene, AP-1 transcription factor subunit	DNA-binding	−6.702

**Table 2:** A list of 30 representative upstream regulators of Fer-1 treated AD aorta.

Upstream Regulator	Molecule Type	Predicted Activation State	Activation z-Score	*p*-Value of Overlap	Target Molecules in Dataset
*MEF2C*	Transcription regulator	Inhibited	−4.358	3.06 × 10^−18^	*ABRA*, *ACTA1*, *ACTN2*, *ATP2A1*, *Ccl9*, *CKM*, *COL10A1*, *CXCL2*, *CXCL6*, *FOSB*, *IL6*, *ITGB1BP2*, *KCNA5*, *KCNJ2*, *LMOD2*, *MMP8*, *MYH1*, *MYH7*, *MYL11*, *MYL2*, *MYOM2*, *MYOT*, *MYOZ1*, *PPARGC1A*, *SMYD1*, *TNNI1*, *TNNI2*, *TNNT1*, *TTN*
*LHX1*	Transcription regulator	Inhibited	−4.199	4.66 × 10^−10^	*ACSM2A*, *ALDOB*, *CLDN2*, *Cyp2j5*, *FUT9*, *GSTA5*, *HNF4A*, *Kap*, *Keg1*, *LRP2*, *LRRC19*, *MEP1A*, *MMP8*, *PAH*, *SLC34A1*, *SLC47A1*, *SLC5A8*, *Sult1d1*, *UMOD*
*GATA1*	Transcription regulator	Inhibited	−3.8	6.01 × 10^−7^	*ALAS2*, *ALOX12*, *CCL5*, *CXCR3*, *EPB42*, *FECH*, *FOSB*, *GATA3*, *GP1BA*, *GP6*, *GP9*, *Gypa*, *HBA1/HBA2*, *HBB*, *Hbb-b1*, *Hbb-b2*, *HDAC11*, *ITGAX*, *MPL*, *NEFH*, *NFE2*, *SLC4A1*, *SNCA*, *SPTA1*, *TUBB1*
*ERK* (family)	Group	Inhibited	−3.774	0.00357	*ARG2*, *Ccl7*, *CSF2*, *CXCL2*, *CXCL6*, *DIO2*, *FOSL1*, *GDF15*, *HAS1*, *HMOX1*, *HOMER1*, *IL1B*, *IL6*, *LIF*, *SERPINB2*
F2	Peptidase	Inhibited	−3.741	0.00927	*Ccl9*, *CXCL3*, *CYP2B6*, *DHRS9*, *EPB41*, *FOSB*, *FOSL1*, *GP6*, *HMOX1*, *IL1B*, *IL6*, *OSM*, *SELE*, *SOD2*, *TFPI2*
Hydrogen peroxide	Chemical–endogenous mammalian	Inhibited	−3.721	0.0384	*AKR1C1/AKR1C2*, *CKMT2*, *CSF3*, *CXCL3*, *CXCL6*, *CXCR3*, *FOSL1*, *GDF15*, *GSTA5*, *HDC*, *HMOX1*, *IL1B*, *IL6*, *MB*, *MIOX*, *MMP8*, *NOS1*, *PAH*, *PPARGC1A*, *SOD2*, *SQLE*, *TCF3*
*TGFB1*	Growth factor	Inhibited	−3.686	8.56 × 10^−5^	*ACTA1*, *ADAMTS4*, *AKR1C1/AKR1C2*, *ALDOB*, *ALOX12*, *ALOX15*, *AMY2B*, *BPIFB1*, *CCL5*, *Ccl7*, *CD40*, *CDHR1*, *Ces2c*, *CHRNB4*, *CKM*, *COL10A1*, *CRHR2*, *CRMP1*, *CSF2*, *CXCL2*, *CXCL3*, *CXCR3*, *DISP2*, *EGF*, *ENO1*, *ENO2*, *F5*, *FOSB*, *FOSL1*, *G6PC1*, *GATA3*, *GDF15*, *GPR158*, *GRIA1*, *GSTA5*, *GZMA*, *H19*, *HAS1*, *HMOX1*, *HNF4A*, *IFI16*, *IL1B*, *IL6*, *ITGAX*, *Kap*, *KCNQ3*, *KNG1*, *KRT14*, *KRT5*, *KRT8*, *L1CAM*, *LDHA*, *LIF*, *MAOA*, *MID1*, *MSTN*, *MUC5B*, *Mx1*, *MYH7*, *MYL11*, *MYL3*, *MYOCD*, *NOS1*, *OSM*, *PAK3*, *PGAM2*, *PNMT*, *PPARGC1A*, *PRSS3*, *RND1*, *RSAD2*, *SCGB1A1*, *SELE*, *SERPINA1*, *SLC4A1*, *SOD2*, *SPOCK1*, *SQLE*, *STAR*, *D4*, *TNFAIP6*, *TNFRSF14*, *TNFSF13B*
Prostaglandin E2	Chemical–endogenous mammalian	Inhibited	−3.527	0.000226	*ADAMTS4*, *ALOX15*, *CCL5*, *CD40*, *CSF3*, *CST7*, *CXCL3*, *Cxcl9*, *CXCR3*, *FOSB*, *FOSL1*, *GBP6*, *H2-M2*, *HDC*, *HMOX1*, *IL1B*, *IL6*, *MYOZ1*, *OSM*, *SCN9A*, *Tcstv4*, *TNFAIP6*, *TREM1*
*AGT*	Growth factor	Inhibited	−3.291	1.01 × 10^−15^	*ACTA1*, *ADAM23*, *ADAMTS4*, *ALOX12*, *ALOX15*, *AMY2B*, *ANXA1*, *ATP6V0A4*, *CAV3*, *CCKAR*, *CCL5*, *Ccl7*, *Ccl9*, *CEL*, *CELA2A*, *CLPS*, *COL10A1*, *CPA1*, *CPA2*, *CTRB2*, *CXCL2*, *CXCL3*, *CYP4A11*, *DBH*, *DCUN1D3*, *DIO2*, *DMBT1*, *FOSL1*, *GABRB3*, *GALNT13*, *GDF15*, *GSTA5*, *HMOX1*, *IFI16*, *IL1B*, *IL6*, *ITGAX*, *KCNJ2*, *L1CAM*, *LIF*, *MAP2*, *MATN2*, *MMP8*, *MYH7*, *NCALD*, *NOS1*, *NPY*, *PM20D1*, *PNLIP*, *PNLIPRP1*, *PNLIPRP2*, *PPARGC1A*, *PRSS2*, *PTGIS*, *REG1A*, *RNASE1*, *SCG2*, *SELE*, *SERPINB2*, *SIGLEC1*, *SLC12A3*, *SLC13A1*, *SLC6A2*, *SNCA*, *SOD2*, *SRGAP3*, *SYT4*, *TH*, *TNFRSF18*, *TTN*, *XIRP1*, *ZG16*
*IL1B*	Cytokine	Inhibited	−3.266	1.8 × 10^−12^	*ADAMTS4*, *ALOX12*, *ALOX15*, *ANXA1*, *BPIFB1*, *CALB1*, *CCKAR*, *CCL5*, *Ccl7*, *Ccl9*, *CD40*, *CD40LG*, *COL10A1*, *CSF2*, *CSF3*, *CXCL2*, *CXCL3*, *CXCL6*, *Cxcl9*, *CYP2B6*, *FOSB*, *FOSL1*, *G0S2*, *GATA3*, *GBP6*, *GDF15*, *GRIA1*, *H19*, *HAS1*, *HLA-E*, *HMOX1*, *HNF4A*, *HOMER1*, *IFI16*, *IL1B*, *IL6*, *INSRR*, *ISG20*, *ISL1*, *ITGB8*, *KRT5*, *LDHA*, *LIF*, *MMP8*, *MUC5B*, *NOS1*, *NPY*, *NTRK1*, *OSM*, *PCSK1*, *PPARGC1A*, *Ppbp*, *PSMB9*, *PTGIS*, *RAB3C*, *RNASE1*, *RSAD2*, *SAA1*, *SCGB1A1*, *SCN9A*, *SELE*, *SERPINB2*, *SMAD9*, *SNAP25*, *SNCA*, *SOD2*, *STMN2*, *TFPI2*, *TNFAIP6*, *TNFSF13B*, *TOB1*, *TREM1*, *UBE2L6*
*NFAT5*	Transcription regulator	Inhibited	−3.18	9.68 × 10^−5^	*ACTN2*, *CSF2*, *CSF3*, *CXCL3*, *Cxcl9*, *IFI16*, *IL1B*, *IL6*, *LIF*, *Mx1*, *Oas1c (includes others)*, *PLIN2*, *PTGIS*, *RSAD2*
*REST*	Transcription regulator	Inhibited	−3.177	8.68 × 10^−12^	*CHGB*, *FUT9*, *GRIA2*, *KCNQ2*, *KCNQ3*, *KIAA1549L*, *L1CAM*, *NCAM2*, *NEFH*, *Nefm*, *PCLO*, *PCSK1*, *PPARGC1A*, *SCG2*, *SCG3*, *SLC13A1*, *SLC7A14*, *SNAP25*, *SRRM4*, *STMN2*, *STMN3*, *SYT1*, *SYT4*, *TNNT1*
E. coli B4 lipopolysaccharide	Chemical toxicant	Inhibited	−3.174	1.06 × 10^−6^	*ANXA1*, *CCL5*, *CD40*, *CD40LG*, *Cd52*, *CSF2*, *CSF3*, *CXCL2*, *CXCL3*, *CXCL6*, *F5*, *FOSB*, *HMOX1*, *IFI16*, *IL1B*, *IL6*, *Mx1*, *RSAD2*, *SELE*, *SNCA*, *SOD2*, *Stfa2/Stfa2l1*, *TH*, *TNFSF8*
Cholesterol	Chemical–endogenous mammalian	Inhibited	−3.124	0.000116	*ACACA*, *Ccl7*, *CXCL2*, *CXCL3*, *DDC*, *GP1BA*, *GSTA5*, *IL1B*, *IL6*, *ITGAX*, *KCNA5*, *MAP2*, *MMP8*, *PLIN2*, *PPARGC1A*, *SAA1*, *SQLE*, *STARD4*
*RELA*	Transcription regulator	Inhibited	−3.002	2.39 × 10^−5^	*ALPK2*, *ANKRD2*, *BEX1*, *CCL5*, *CD40*, *CSF2*, *CXCL2*, *CXCL3*, *CXCL6*, *CYP2B6*, *DIO2*, *FOSB*, *GDF15*, *HMOX1*, *HNF4A*, *IL1B*, *IL6*, *ISG20*, *KRT8*, *Madcam1*, *Mx1*, *NEFH*, *PSMB9*, *REG3G*, *SAA1*, *SCN9A*, *SELE*, *SOD2*, *TFPI2*, *TREM1*
SP2509	Chemical reagent	Activated	3.939	0.0116	*CCL5*, *CNTN1*, *CNTNAP2*, *CRMP1*, *Cxcl9*, *IGF2BP2*, *ISL1*, *KIF5C*, *L1CAM*, *MAP2*, *NEFL*, *NRXN1*, *PAK3*, *SPOCK1*, *STMN2*, *SYT1*
SB203580	Chemical drug	Activated	3.604	8.52 × 10^−8^	*ADAMTS4*, *ANXA1*, *BPGM*, *CAV3*, *CCL5*, *Ccl7*, *CD40*, *CSF2*, *CST7*, *CXCL3*, *Cxcl3*, *EGF*, *ENO1*, *FOSB*, *G6PC1*, *HAS1*, *HMOX1*, *HPCA*, *IL1B*, *IL6*, *ISG20*, *MMP8*, *MSTN*, *MUC5B*, *MYL11*, *MYOCD*, *NEFH*, *PPARGC1A*, *RND1*, *SELE*, *SMYD1*, *SOD2*, *TFPI2*, *TNFAIP6*, *TNFSF8*, *TREM1*
*KDM5A*	Enzyme	Activated	3.441	3.71 × 10^−9^	*ACTN2*, *Actn3*, *CACNA1S*, *HMOX1*, *HOMER1*, *MYBPC1*, *MYH2*, *MYH4*, *MYH7*, *MYOM2*, *PGAM2*, *REEP1*, *RYR1*, *SOD2*, *TCAP*, *TNNC2*, *TNNI2*, *TNNT1*, *TRIM72*
SP600125	Chemical drug	Activated	3.315	0.0023	*ADAMTS4*, *CCL5*, *CSF2*, *CSF3*, *CXCL2*, *CXCL3*, *CXCL6*, *FOSB*, *HAS1*, *HMOX1*, *HNF4A*, *IL1B*, *IL6*, *MUC5B*, *MYH7*, *Oas1c (includes others)*, *SELE*, *SOD2*, *TNFRSF18*
*FBXO32*	Enzyme	Activated	3.24	5.81 × 10^−8^	*CSF2*, *CXCL2*, *CXCL3*, *Cxcl3*, *CXCL6*, *IL1B*, *IL6*, *MYOCD*, *PDIA2*, *PTGIS*, *REG3G*, *SERPINB2*, *SOD2*, *TFPI2*
N-acetyl-L-cysteine	Chemical drug	Activated	3.185	0.00919	*ACTA1*, *CCL5*, *CXCL3*, *HDC*, *HMOX1*, *IL1B*, *IL6*, *MIOX*, *MYOCD*, *PPARGC1A*, *SELE*, *SNCA*, *SOD2*, *TRIM63*
IMMUNOGLOBULIN (complex)	Complex	Activated	2.842	1.6 × 10^−10^	*ACACA*, *ACTA1*, *ALAS2*, *APOL2*, *BC147527*, *BPGM*, *CCL5*, *Ccl7*, *Ccl9*, *CD40*, *CD40LG*, *Cd52*, *CHST2*, *CSF2*, *CST7*, *CXCL2*, *CXCL3*, *CXCL6*, *Cxcl9*, *CXCR3*, *ENO1*, *ENO2*, *FAIM2*, *FOSB*, *G6PC1*, *GATA3*, *GZMA*, *HEMGN*, *HLA-E*, *HMOX1*, *IFI16*, *IL1B*, *IL6*, *ISG20*, *ITGAX*, *ITGB8*, *JCHAIN*, *MFSD2A*, *MPO*, *Mx1*, *MZB1*, *NEB*, *NOS1*, *OSM*, *PGAM2*, *PPARGC1A*, *PRDX5*, *PYGM*, *RHAG*, *RSAD2*, *SCG2*, *SELE*, *SLC25A37*, *SOD2*, *SQLE*, *TIGIT*, *TNFRSF18*, *TNFSF13B*, *TNFSF8*, *TTN*, *UGP2*
*ARID1A*	Transcription regulator	Activated	2.813	0.00079	*ANXA1*, *CAV3*, *CKM*, *CXCL2*, *GATA3*, *IL1B*, *KRT14*, *KRT5*, *KRT8*, *SERPINA1*
*ALPHA CATENIN* (family)	Group	Activated	2.81	0.0185	*ADAMTS4*, *CXCL2*, *CXCL6*, *IL1B*, *IL6*, *SAA1*, *SELE*, *TNFAIP6*
*LTBR*	Transmembrane receptor	Activated	2.789	4.85 × 10^−5^	*CCL21*, *CSF2*, *GPM6B*, *IL6*, *Klra4 (includes others)*, *Madcam1*, *SERPINA1*, *TNFSF13B*
Diphenyleneiodonium	Chemical reagent	Activated	2.759	0.000457	*CXCL2*, *CXCL3*, *GDF15*, *HMOX1*, *IL1B*, *IL6*, *SELE*, *SOD2*
*PTF1A*	Transcription regulator	Activated	2.728	7.66 × 10^−8^	*AMY2B*, *CACNA2D3*, *CEL*, *CPA1*, *CPA2*, *CTRB2*, *GRIK2*, *ISL1*, *KLHL14*, *NPY*, *PRSS3*, *TFAP2B*
Sb202190	Chemical drug	Activated	2.671	0.00141	*CAV3*, *Ccl9*, *CKM*, *CSF2*, *HAS1*, *HMOX1*, *IL1B*, *IL6*, *PPARGC1A*, *SCN9A*, *SELE*
*TP53*	Transcription regulator	Activated	2.654	0.0468	*ADGRB3*, *ALDOC*, *ALOX15*, *ANXA1*, *APOBEC2*, *CCL5*, *CEL*, *CKM*, *CKMT2*, *CSF2*, *CSMD3*, *CXCL2*, *CXCL3*, *DHRS9*, *DNASE1*, *EGF*, *ENO2*, *ESRRB*, *F5*, *FOSL1*, *G0S2*, *G6PC1*, *GDF15*, *GPM6B*, *H19*, *HDC*, *HMOX1*, *IFI16*, *IGF2BP2*, *IL1B*, *IL6*, *ITGB1BP2*, *KCNJ2*, *KRT14*, *KRT8*, *LDHA*, *LIF*, *LRAT*, *MB*, *MRPS2*, *Mx1*, *MYL2*, *MYL3*, *MYOCD*, *NFE2*, *NOS1*, *NR2F1*, *NRAP*, *PAK3*, *PGAM2*, *PPARGC1A*, *PSMB9*, *SCN3B*, *SELE*, *SERPINB2*, *SLC5A8*, *SOD2*, *SQLE*, *SRGAP3*, *STARD4*, *TCAP*, *TFPI2*, *TMOD4*, *TNFRSF18*, *TOB1*, *TTN*
*NR5A2*	Ligand-dependent nuclear receptor	Activated	2.607	6.61 × 10^−5^	*Ccl7*, *CEL*, *CELA3B*, *CPA1*, *CPA2*, *CTRL*, *Cxcl9*, *HNF4A*, *IL1B*, *IL6*, *PNLIP*, *SAA1*, *SYCN*

## Data Availability

All data presented in the present study are available from the corresponding author on reasonable request.

## Supplementary Materials

The following supporting information can be downloaded at: https://www.mdpi.com/article/10.3390/ijms26094338/s1.

## Abbreviations

AD	Aortic Dissection
Fer-1	Ferrostatin-1
SMC	Smooth Muscle Cell
ECM	Extracellular Matrix
BAPN	β-aminopropionitrile
Ang-II	Angiotensin II
SPF	Specific Pathogen-free
IPA	Ingenuity Pathway Analysis
PCA	Principal Component Analysis
DEGs	Differentially Expressed Genes
FC	Fold Change
