# Supplementary material for: Transcriptome Insights into Protective Mechanisms of Ferroptosis Inhibition in Aortic Dissection
Source: Int J Mol Sci. 2025 May 2;26(9):4338. doi: 10.3390/ijms26094338 (PMC12072690; doi:10.3390/ijms26094338)
Supplement: Supplementary file 1 [file ijms-26-04338-s001.zip › Supplemental figures with legends.pdf]

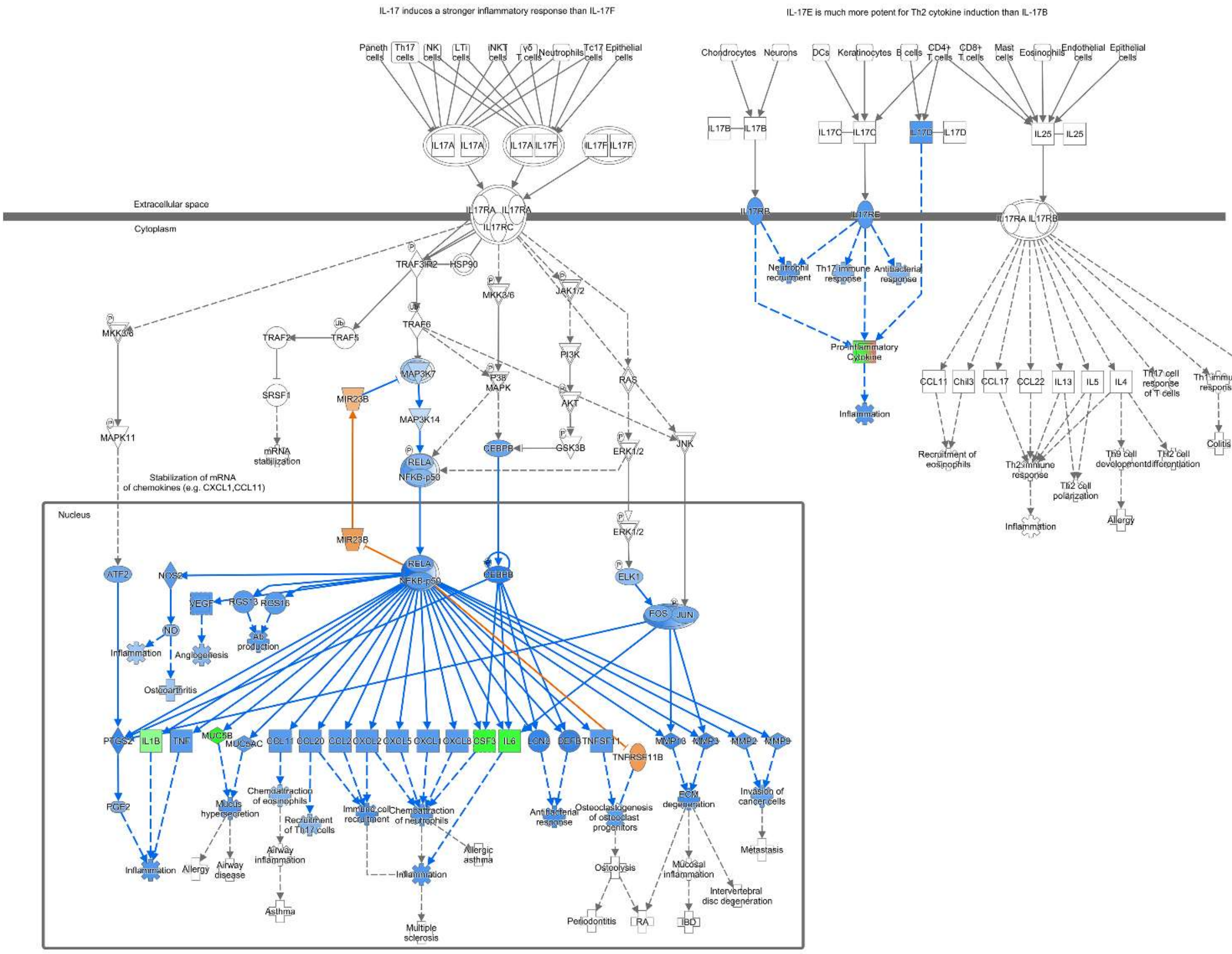

Figure S1. Experiment validation of Ingenuity Pathway Analysis (IPA). Canonical pathway analysis in IL-17 signaling pathway. Molecules highlighted in the pathway map represent differentially expressed genes (DEGs) mapped to the pathway. Orange indicates predicted activation; blue indicates predicted inhibition. Red nodes represent increased gene expression in the dataset, while green nodes represent decreased expression.

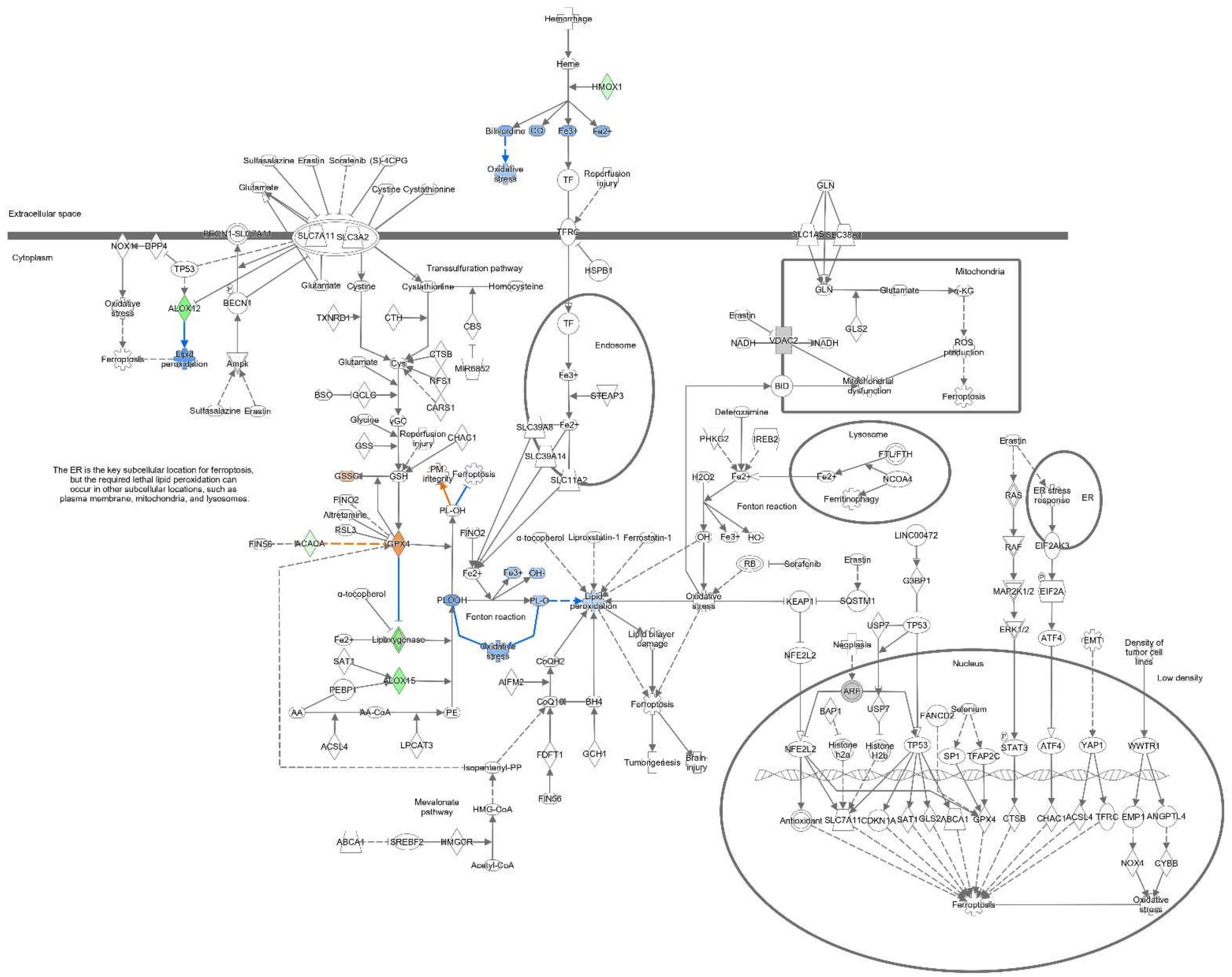

Figure S2. Experiment validation of Ingenuity Pathway Analysis (IPA). Canonical pathway analysis in ferroptosis signaling pathway Molecules highlighted in the pathway map represent differentially expressed genes (DEGs) mapped to the pathway. Orange indicates predicted activation; blue indicates predicted inhibition. Red nodes represent increased gene expression in the dataset, while green nodes represent decreased expression.

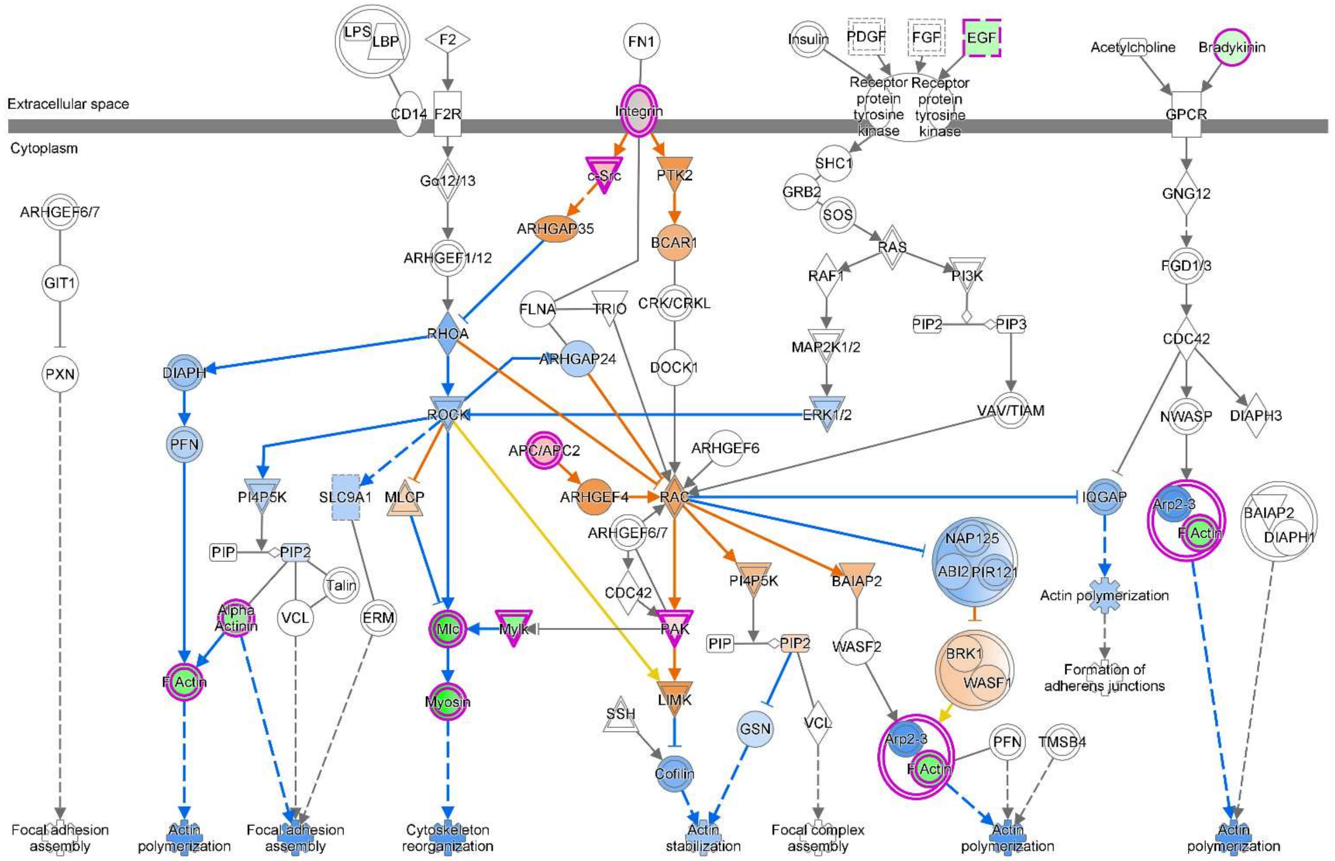

Figure S3. Experiment validation of Ingenuity Pathway Analysis (IPA). Canonical pathway analysis in actin cytoskeleton signaling pathway. Molecules highlighted in the pathway map represent differentially expressed genes (DEGs) mapped to the pathway. Orange indicates predicted activation; blue indicates predicted inhibition. Red nodes represent increased gene expression in the dataset, while green nodes represent decreased expression.

S

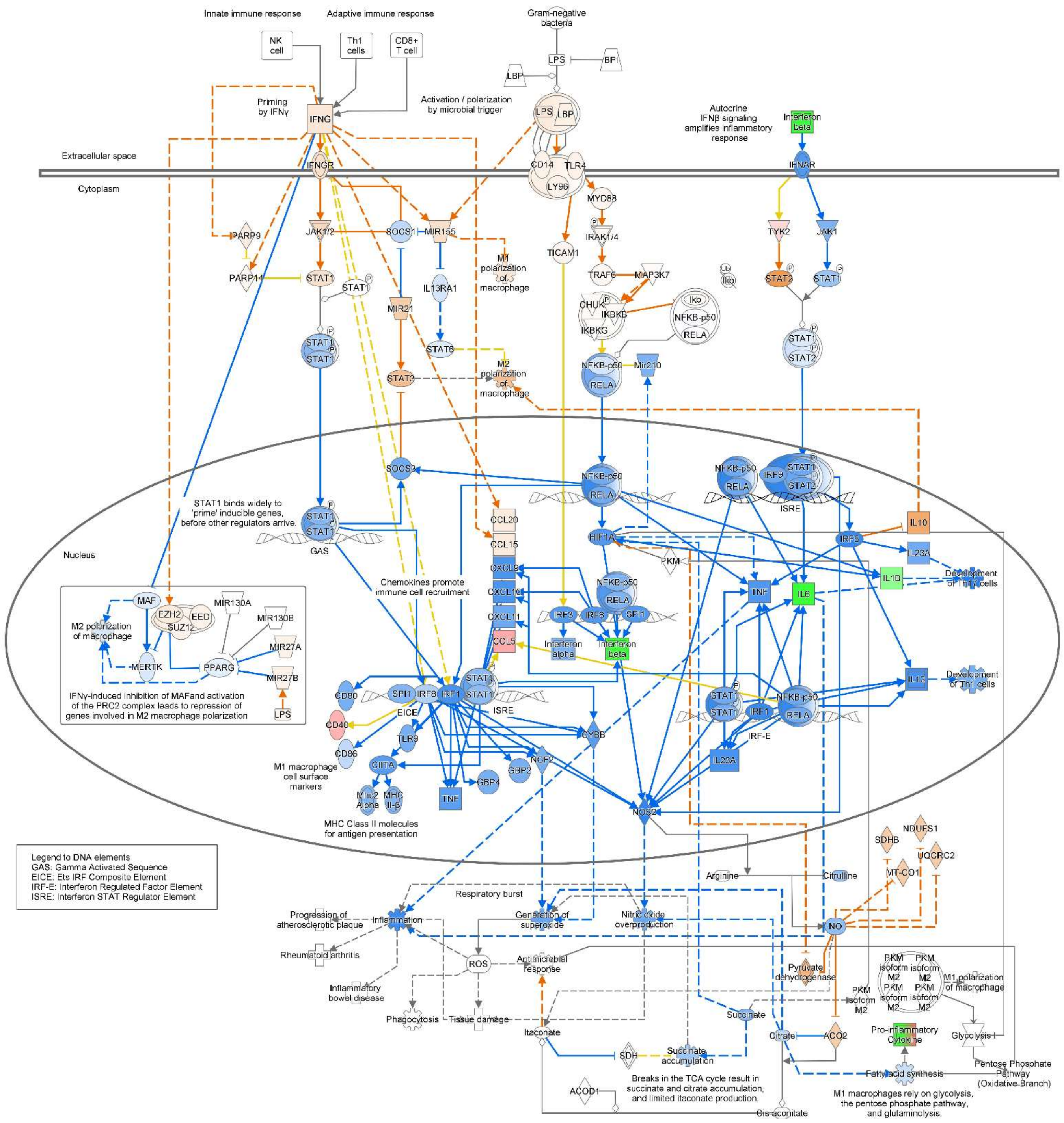

Figure S4. Experiment validation of Ingenuity Pathway Analysis (IPA). Canonical pathway analysis in macrophage classical activation signaling pathway. Molecules highlighted in the pathway map represent differentially expressed genes (DEGs) mapped to the pathway. Orange indicates predicted activation; blue indicates predicted inhibition. Red nodes represent increased gene expression in the dataset, while green nodes represent decreased expression.

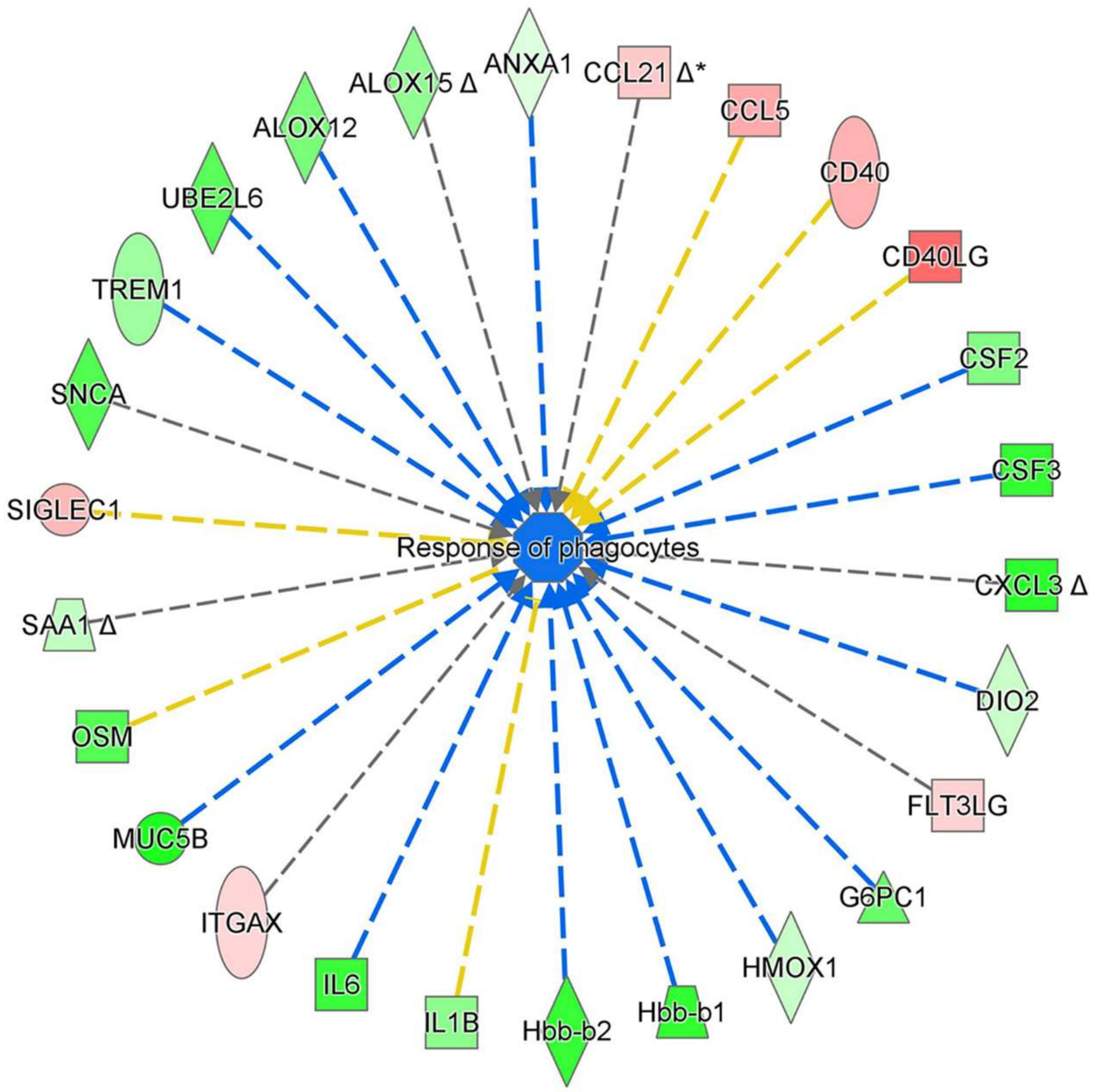

Figure S5. Differentially expressed genes associated with phagocyte responses in Fer-1–treated aortic tissue. The subset of differentially expressed genes (DEGs) identified from RNA-seq data that are associated with the "response of phagocytes" biological function. Red nodes represent increased gene expression in the dataset, while green nodes represent decreased expression.

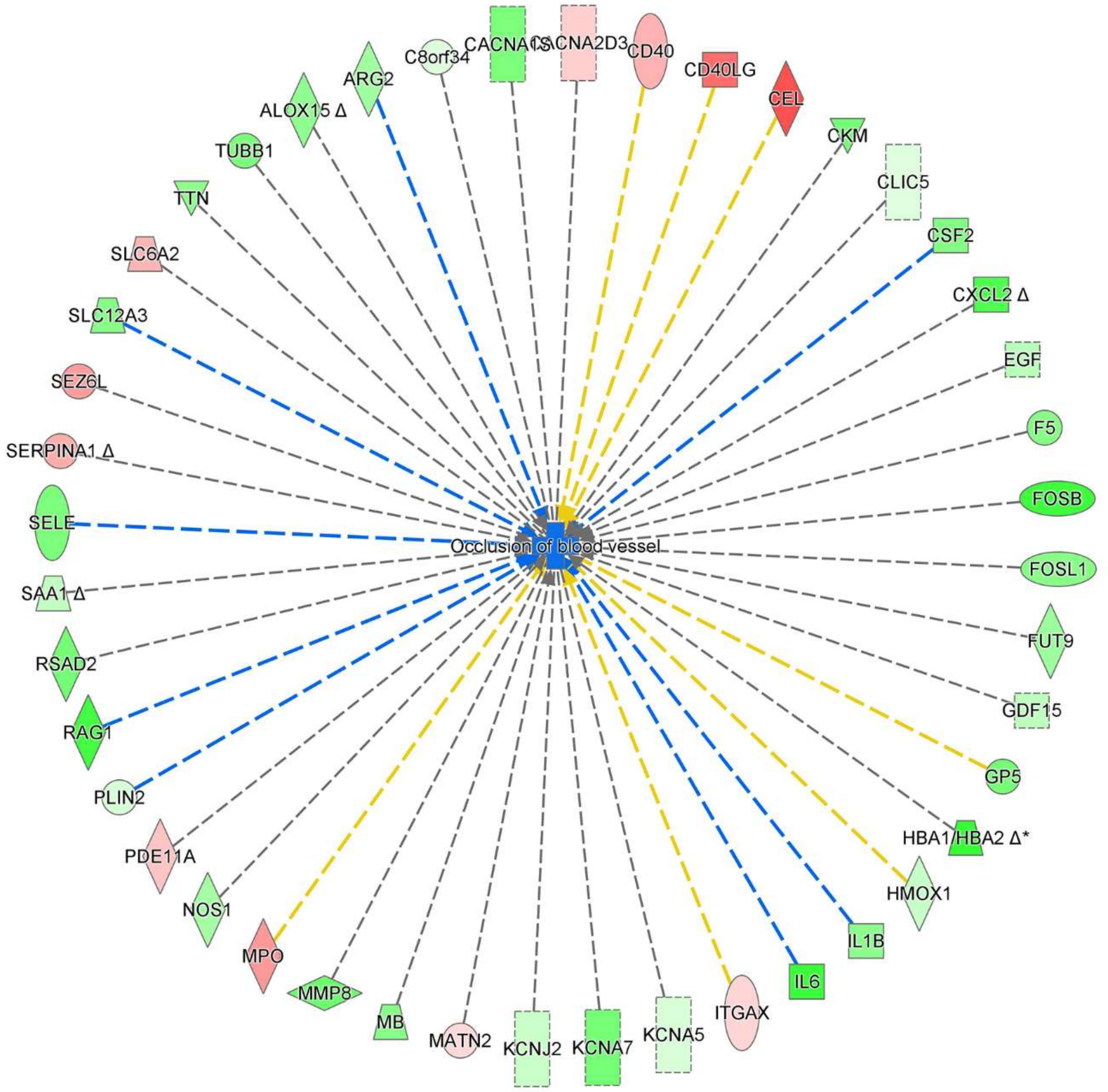

Figure S6. Differentially expressed genes associated with occlusion of blood vessel in Fer-1–treated aortic tissue. The subset of differentially expressed genes (DEGs) identified from RNA-seq data that are associated with the “occlusion of blood vessel” biological function. Red nodes represent increased gene expression in the dataset, while green nodes represent decreased expression.

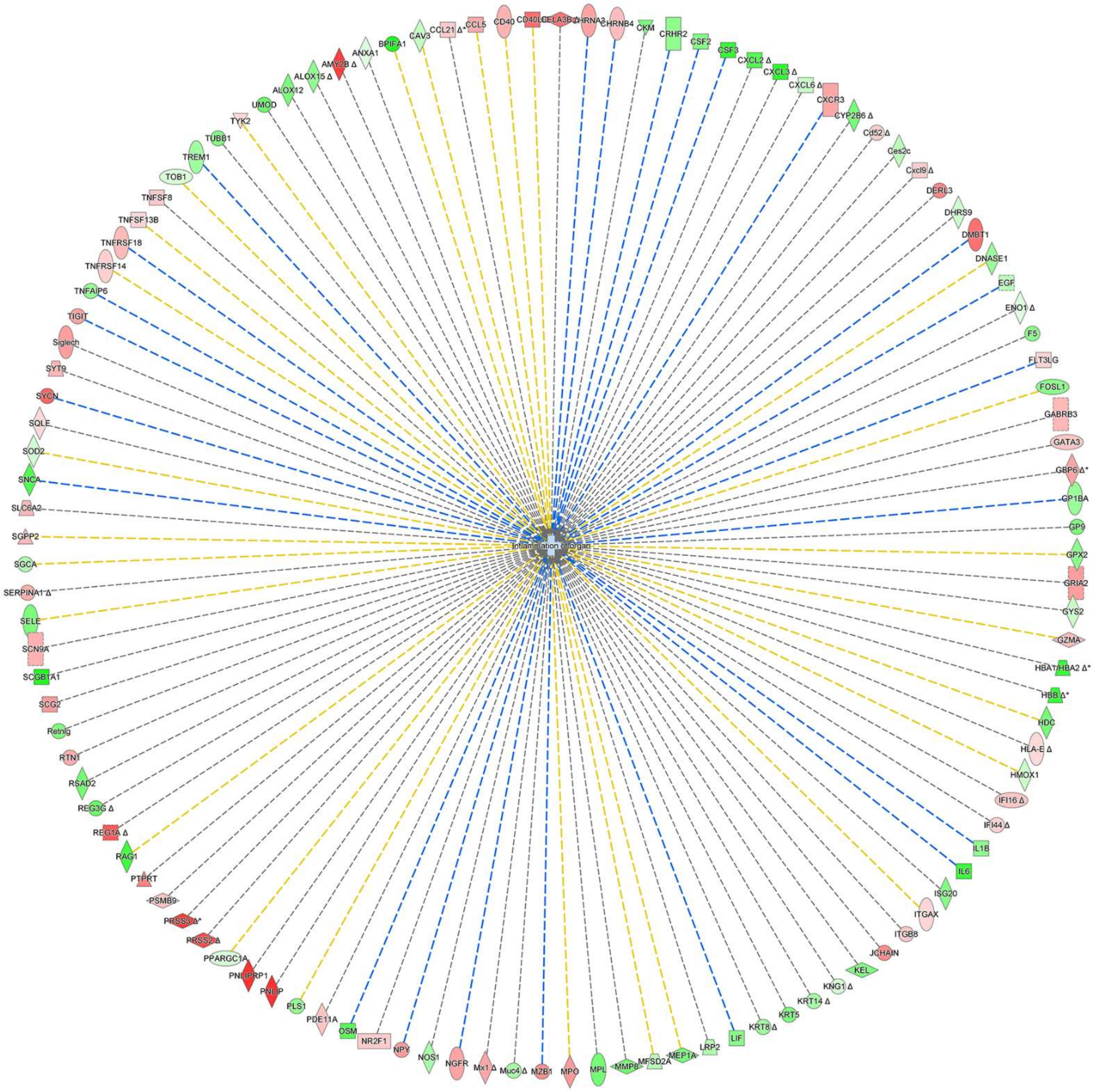

Figure S7. Differentially expressed genes associated with organ inflammation in Fer-1–treated aortic tissue. The subset of differentially expressed genes (DEGs) identified from RNA-seq data that are associated with the “inflammation of organ” biological function. Red nodes represent increased gene expression in the dataset, while green nodes represent decreased expression.

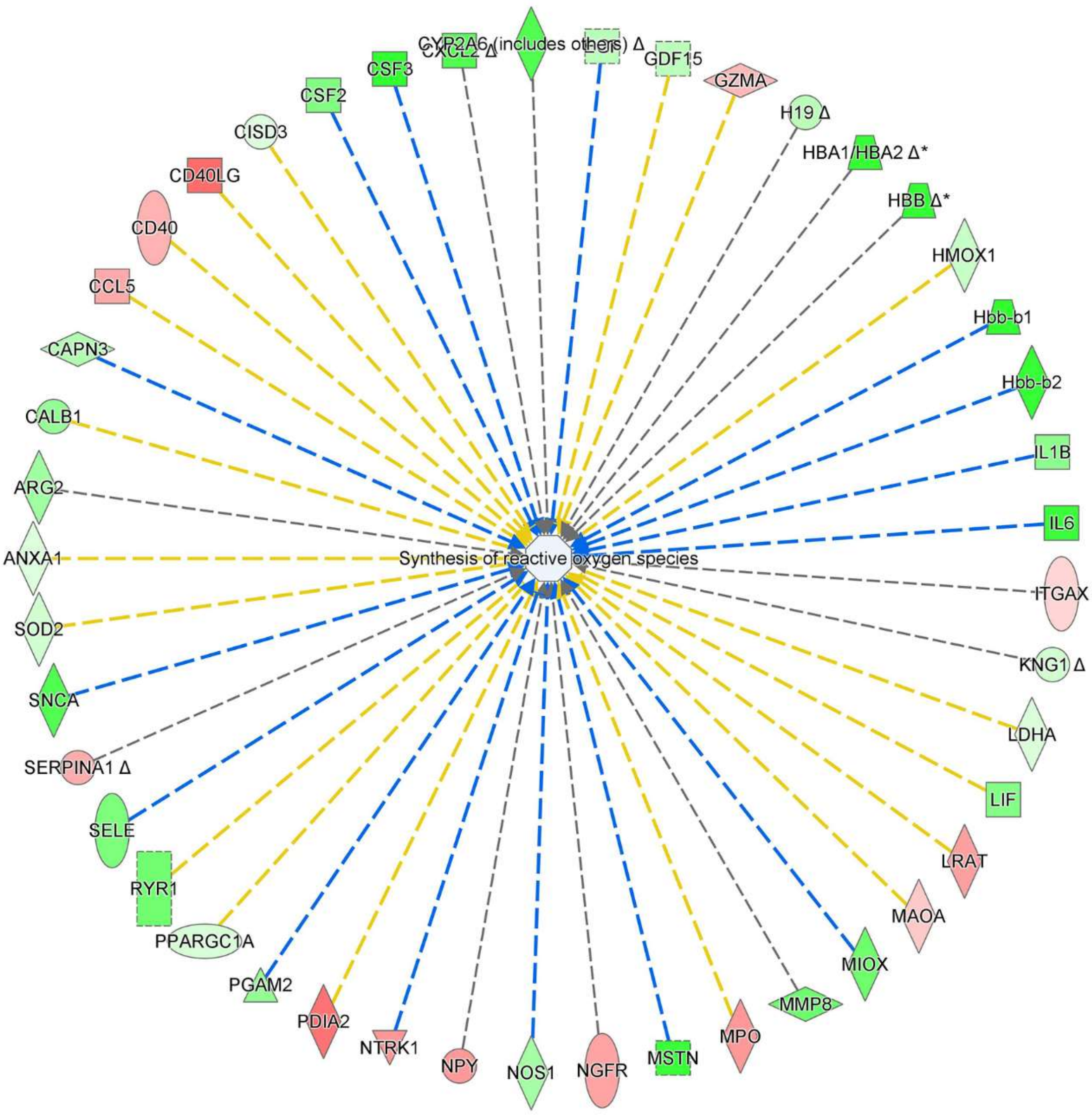

Figure S8. Differentially expressed genes associated with ROS synthesis in Fer-1–treated aortic tissue. The subset of differentially expressed genes (DEGs) identified from RNA-seq data that are associated with the "synthesis of ROS" biological function. Red nodes represent increased gene expression in the dataset, while green nodes represent decreased expression.
